# The Changes in Fecal Bacterial Communities in Goats Offered Rumen-Protected Fat

**DOI:** 10.3390/microorganisms12040822

**Published:** 2024-04-19

**Authors:** Hu Liu, Weishi Peng, Kaiyu Mao, Yuanting Yang, Qun Wu, Ke Wang, Meng Zeng, Xiaotao Han, Jiancheng Han, Hanlin Zhou

**Affiliations:** Zhanjiang Experimental Station, Chinese Academy of Tropical Agricultural Sciences, Zhanjiang 524000, China; liuh2018@lzu.edu.cn (H.L.); m17378095519@163.com (W.P.); mmiaozzi@163.com (K.M.); ytyang10@163.com (Y.Y.); wuqun.2006@163.com (Q.W.); zmeng0909@163.com (M.Z.);

**Keywords:** rumen-protected fat, goat, fecal bacteria communities

## Abstract

Leizhou goats are famous for their delicious meat but have inferior growth performance. There is little information on rumen-protected fat (RPF) from the Leizhou goat. Hence, we observed the effects of RPF on growth, fecal short-chain fatty acids, and bacteria community with respect to Leizhou goats. Twelve goats (13.34 ± 0.024 kg) were selected and assigned randomly to one of two treatments: (1) a control diet (CON) and (2) 2.4% RPF with a control diet (RPF). The final body weight and average daily gain (ADG) were greater (*p* < 0.05), and the dry matter intake (DMI): ADG was lower (*p* < 0.05) in the RPF group than in the CON group. There were no differences in DMI between the CON and RPF groups. The concentrations of total short-chain fatty acids, acetate, propionate, and butyrate were lower (*p* < 0.05) in the RPF group than in the CON group. The relative abundances of *Ruminococcus*, *Rikenellaceae_RC9_gut_group*, *Treponema*, *norank_f__norank_o__RF39*, *Eubacterium_siraeum_group*, and *Ruminococcus_torques_group* were lower (*p* < 0.05) in the RPF group than in the CON group. The relative abundances of *Bacteroides*, *norank_f__norank_o__Clostridia_UCG-014*, *norank_f__Eubacterium_coprostanoligenes_group*, *Eubacterium_ruminantium_group*, *norank_f__Oscillospirale-UCG-010*, *Oscillospiraceae_UCG-002*, and *Family_XIII_AD3011_group* were greater (*p* < 0.05) in the RPF group than in the CON group. It was concluded that RPF could improve the goats’ growth performance by regulating their fecal bacteria communities.

## 1. Introduction

The indigenous Leizhou goat, also called the Hainan Black goat, was raised in the south of China (such as the Leizhou Peninsula and Hainan Island) and grazes in humid regions. In addition, it is the only dominant goat breed from Guangdong Province in the Catalogue of National Livestock and Poultry Genetic Resources of China [1]. Leizhou goats, with a population of 1 million, are vital for the livelihoods of local farmers and famous for their high-quality meat [2]. However, production efficiency for this goat is very low due to the insufficient forage availability between November and March in the next year. Hence, it is urgent to improve production efficiency for the Leizhou goat.

With regard to improving ruminant production, numerous studies have reported that supplementary amino acids or fatty acids could improve production for yaks, sheep, and goats [3,4,5]. Fat usage has become a common strategy for providing energy in animal production. However, fat is degraded by rumen microorganisms, whereas rumen-protected fat (RPF) can bypass the rumen and move to the small intestine, increasing the utilization of this fat. Numerous studies on RPF have focused on dairy cows [6], beef cattle [7], goats [5], and sheep [8], but there is little information on the Leizhou goat.

As gut microorganisms, bacteria play a crucial role in a variety of physiological processes in ruminants, such as nutrient metabolism, immune protection, homeostasis, and body development. Previous studies showed that rumen-protected products could change the bacteria communities in ruminants [9,10]. It was reported that a lower ratio of Firmicutes to Bacteroidetes was linked to an inhibition of fat deposition and a decreased ADG of the host animal [11]. In addition, the abundance of *Porphyromonadaceae bacterium DJF B175* increased in the high-average-daily-gain group, while that of Lactobacillus reuteri decreased in pre-weaned beef calves [12]. However, there has been little research on changes in the gut microbiota with respect to fatty acids. Consequently, the objective of this experiment was to fill this gap by examining the effect of RPF on the growth performance and fecal bacteria communities of the Leizhou goat, providing a potential strategy for improving animal growth performance and increasing profits when raising Leizhou goats intensively.

## 2. Materials and Methods

This experiment was carried out from October to December 2023 at Zhanjiang Experiments Station of the Chinese Academy of Tropical Agricultural Sciences (21°16′12″ N, 110°21′27″ E), Zhanjiang City, Guangdong Province, China. All protocols and experimental procedures were approved by the Animal Care and Use Committee of Zhanjiang Experiments Station of the Chinese Academy of Tropical Agricultural Sciences (Protocol number: ZES 202306010).

### 2.1. Animals, Design, and Diets

Twelve goats (13.34 ± 0.024 kg), all of which were castrated males and 6 months of age, were held individually in cages (0.8 m × 1.2 m) equipped with feeders and automatic waterers. Within each block, the goats were assigned randomly to one of two treatments: (1) a control diet (CON) or a (2) 2.4% RPF with control diet. The RPF (consisting of 48% C16:0, 5% C18:0, 36% C18:1, 9% C18:2, and 2% C14:0) supplements were used according to the manufacturer’s recommendations (Yihai Kerry Arawana Holdings Co., Ltd., Shanghai, China). The goats were fed ad libitum a control diet of 500 g/kg of forage and 500 g/kg of concentrate on a dry-matter basis (Table 1; Guangxi Maosen Nongmu Co., Ltd., Nanning City, China).

The feedlot period lasted 56 days, comprising 14 days for diet and cage adaptation and 42 days for the experimental period. The average daily gain (ADG) was calculated throughout the experimental period. Feed was weighted and offered twice daily at 08:00 and 17:00 h. Orts were weighed daily, and DMI was calculated based on the records of the feed offered daily and the weight of the feed remaining the next day. The feed efficiency was calculated as the ratio of ADG to DMI.

### 2.2. Procedures and Sample Collection

Approximately 100 g of diet was collected daily between day 53 to 56. Before morning feeding on day 56, 20 g rectal fecal samples were taken from the animals and then loaded into 25 mL centrifuge tubes (Corning, Shanghai, China). All samples were immediately frozen after collection in liquid nitrogen and stored at −80 °C to analyze bacterial communities and short-chain fatty acids.

### 2.3. Feed Sample Analysis

Feed samples were dried at 65 °C in a forced-air oven for 80 h and then ground through a 1 mm sieve. The dry matter (method 925.45), organic matter (method 990.03), and ether extract (method 920.29) proportions were determined according to the Association of Official Analytical Chemists [13]. The Kjedahl method was applied to determine total nitrogen content of feed using a nitrogen analyzer, and the crude protein content was calculated as follows: total nitrogen × 6.25. The neutral detergent fiber and acid detergent fiber proportions were measured using an automatic fiber analyzer according to Robertson [14] and Van Soest et al. [15], respectively.

### 2.4. Fecal Short-Chain Fatty Acids Analysis

The SCFA concentrations in the stool samples were measured using gas chromatography (GC) via a capillary column (AT-FFAP: 30 m × 0.32 mm × 0.5 μm) using a Shimadzu GC-2010 plus system with a flame ionization detector (Shimadzu Corporation, Kyoto, Japan) according to the method reported by Liu et al. [3] with a minor revision. A 1.000 g fresh feces sample was vortex-mixed with 4 mL of ultrapure water, shaken (Promax 2020; Heidolph Instruments GmbH CO. KG, Shanghai, China) at 4 °C for 3 h, and then centrifuged (15,000 rpm; HT190R, Hunan Xiangyi Laboratory Instrument Development Co., Ltd., Changsha, China) at 4 °C for 15 min. Subsequently, the supernatant (1000 μL) was mixed with 200 μL of metaphosphoric acid solution (25%; Shanghai Macklin Biochemical Technology Co., Ltd., Shanghai, China) for 30 min, centrifuged (15,000 rpm) at 4 °C for 15 min, and finally filtered through a 0.22 μm filter membrane. Aliquots of the supernatants (800 μL) were pipetted into a glass gas chromatography vial to make preparations for the following GC instrument analysis. Briefly, the temperature of the flame ionization detector was set to 280 °C. The carrier gas was highly purified N_2_ (99.99%) with a flow rate of 0.8 mL/min. The SCFA concentrations in fecal samples were determined by comparing them with the standard curve.

### 2.5. DNA Extraction, 16S rRNA Gene Amplification, and Sequencing

The total genomic DNA of fecal bacteria was extracted from 1.00 g samples by using a commercial fecal DNA extraction kit (DP328, Tiangen Biotech, Beijing, China), which was used in accordance with the manufacturer’s instructions. The DNA concentrations and purity were determined using NanoDrop One (Thermo Fisher Scientific, Madison, WI, USA). The quality of the extracted DNA was tested using 1% agarose gel electrophoresis (Axygen Biosciences, Union City, CA, USA). The samples with a purity of 1.8 and above were used for further processing in PCR protocols.

The conventional polymerase chain reaction amplification and bioinformatics analysis of extracted DNA samples were conducted by Shanghai Majorbio Bio-Pharm Technology Co., Ltd. (Shanghai, China). The 16S rRNA gene hypervariable regions V3–V4 were used to identify bacteria and amplified using primers 338F (5′-ACTCCTACGGGAGGCAGCAG-3′) and 806R (5′-GGACTACHVGGGTWTCTAAT-3′). The bacterial 16S amplification and the quality filtering, clustering, and analysis of the 16S rRNA sequencing data were conducted in accordance with Liu et al.’s approach [11]. The reaction conditions and procedures of the PCR amplification of the 16S rRNA gene were as follows: initial denaturation at 95 °C for 3 min, followed by 30 cycles, including denaturation at 95 °C for 30 s, annealing at 55 °C for 30 s, and extension at 72 °C for 30 s, with a final extension at 72 °C for 10 min and holding at 10 °C. The PCR mixtures were prepared in triplicate in 20 μL volumes, which consisted of 4 μL of 5 × TransStart FastPfu buffer, 2 μL of 2.5 mM deoxyribonucleotides triphosphate (dNTPs), 0.8 μL of forward primer (5 mM), 0.8 μL of reverse primer (5 mM), 0.2 μL of bovine serum albumin, 0.4 μL of TransStart FastPfu DNA Polymerase, 10 ng of template DNA, and ddH_2_O added until 20 μL was reached. Agarose gel (2.0%) electrophoresis (Axygen Biosciences, Union City, CA, USA) was applied to assess the success of PCR reactions.

After amplification, purified amplicons were pooled equimolarly and paired-end sequenced (2 × 300 bp) using an Illumina MiSeq PE300 platform (Illumina, San Diego, CA, USA) by Majorbio Bio-Pharm Technology Co., Ltd. (Shanghai, China). Data were analyzed using the free online Majorbio Cloud Platform (www.Majorbio.com, accessed on 26 March 2024).

### 2.6. Statistical Analyses

The data on growth performance and short-chain fatty acids was obtained using SAS software (SAS 9.4, SAS institute Inc., Cary, NC, USA). Results are expressed as the means ± standard error (SEM). Multiple-comparison *p*-values were adjusted using the *t*-test. Statistical significance was set at *p*-values < 0.05.

## 3. Results

### 3.1. Growth Performance

As designed, there were no differences (*p* > 0.05) in initial body weight between the CON and RPF groups (Table 2). The FBW and ADG were greater (*p* < 0.05) and the DMI: ADG was lower (*p* < 0.05) in the RPF group compared to those in the CON group. There were no differences in DMI between the CON and RPF groups.

### 3.2. Fecal Short-Chain Fatty Acids

The concentrations of total SCFAs, acetate, propionate, and butyrate were lower (*p* < 0.05) in the RPF group than in the CON group (Table 3). There were no differences in iso-VFAs and the ratio of acetate to propionate (*p* > 0.05) between the RPF group and CON group.

### 3.3. Collective Sequencing Data Summary

A total of 1,007,445 raw reads were generated from the fecal samples, and 984,087 high-quality sequences remained after quality filtering and the removal of chimeric sequences. A total of 3275 OTUs were obtained based on the 97% nucleotide sequence identity among reads.

**Figure 1:**
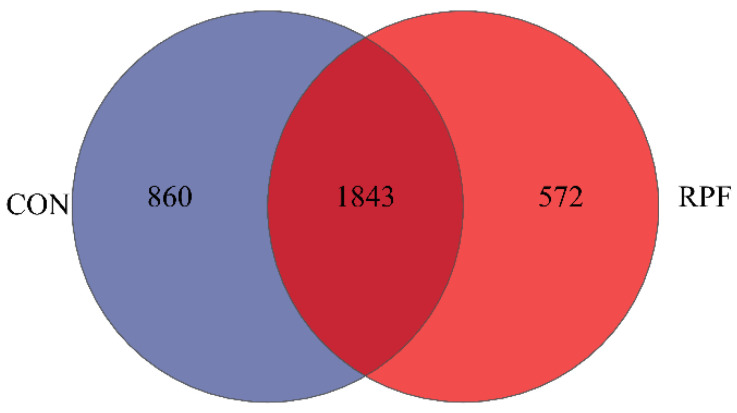
Flower plot showing different and similar OTUs in goats offered rumen-protected fat. CON = control group; RPF = rumen-protected fat.

A total of 1843 OTUs were shared between the CON group and RPF group, accounting for 68.2% and 76.3% of the total OTUs in the CON and RPF groups, respectively (Figure 1). In addition, the number of OTUs specific to the CON and RPF groups were 860 and 572, respectively. There were no differences in ACE, Chao, Shannon, Simpson, and Sobs between the CON group and RPF group (Table 4).

### 3.4. Microbial Community Composition in the Feces

**Figure 2:**
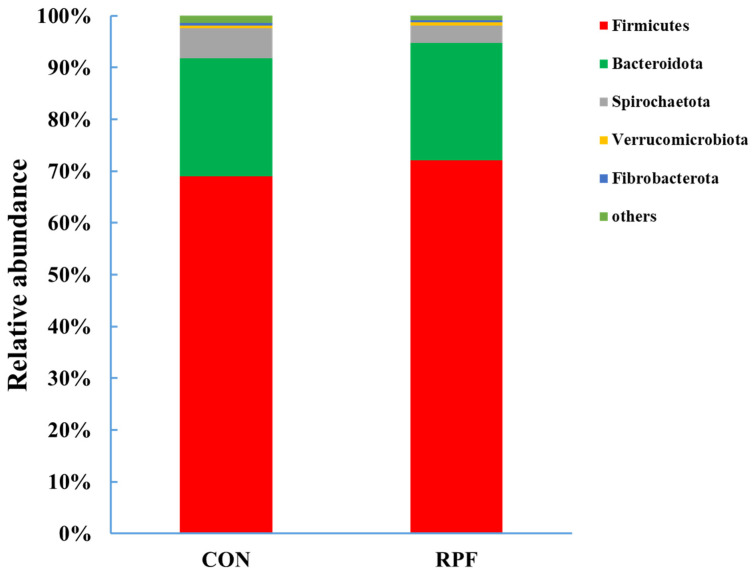
Fecal bacterial relative abundances (at phylum level, >0.5% of total reads) in goats offered rumen-protected fat. CON = control group; RPF = rumen-protected fat.

A total of 15 bacteria phyla were identified in the feces of the CON group and RPF group. The dominant phylum was Firmicutes, accounting for 69.0% and 72.1%, and the second-most-dominant phylum was Bacteroidetes, accounting for 22.9% and 22.6% in the CON group and RPF group, respectively (Figure 2; Table 5). The relative abundances of Firmicutes and Verrucomicrobiota were greater (*p* < 0.05), whereas those of Spirochaetota, Fibrobacterota, and others were lower (*p* < 0.05) in the CON group and RPF group.

**Figure 3:**
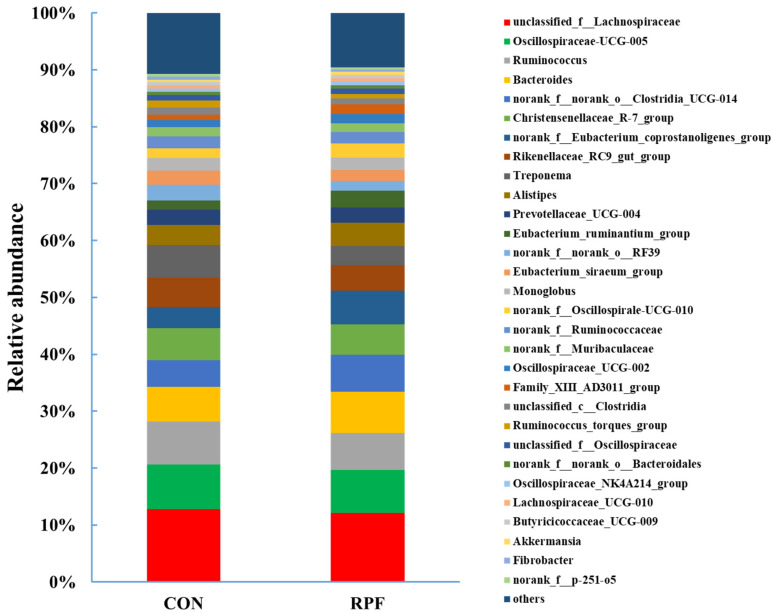
Fecal bacterial relative abundances (at genus level, >0.5% total reads) in goats offered rumen-protected fat. CON = control group; RPF = rumen-protected fat.

A total of 216 bacterial genera were identified in the fecal samples of the two groups of goats. The dominant genus was *unclassified_f_Lachnospiraceae*, accounting for 12.8% and 12.1%, and the second-most-dominant genus was Oscillospiraceae-UCG-005, accounting for 7.84% and 7.55% in the CON and RPF groups, respectively (Figure 3; Table 6). The relative abundances of *Ruminococcus*, *Rikenellaceae_RC9_gut_group*, *Treponema*, *norank_f__norank_o__RF39*, *Eubacterium_siraeum_group*, and *Ruminococcus_torques_group* were lower (*p* < 0.05) in the RPF group than in the CON group. The relative abundances of *Bacteroides*, *norank_f__norank_o__Clostridia_UCG-014*, *norank_f__Eubacterium_coprostanoligenes_group*, *Eubacterium_ruminantium_group*, *norank_f__Oscillospirale-UCG-010*, *Oscillospiraceae_UCG-002*, and *Family_XIII_AD3011_group* were greater (*p* < 0.05) in the RPF group than in the CON group. 

**Figure 4:**
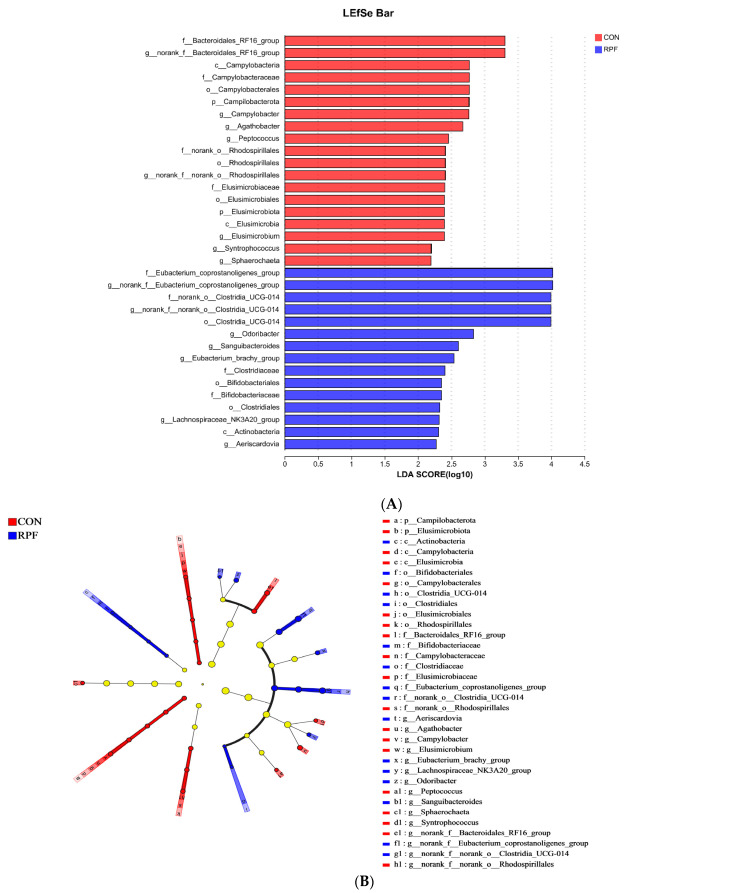
Linear discriminant analysis effect size (LEfSe) results for fecal microbiota in goats consuming diets including rumen-protected fat. (**A**) Linear discriminant analysis. (**B**) Cladogram reported. Prefixes represent abbreviations for the taxonomic rank of each taxon: phylum (p_), class (c_), order (o_), family (f_) and genus (g_). CON = control group; RPF = rumen-protected fat.

Differences in microbiota that varied with RPF supplementation were further identified using linear discriminant analysis effect size (LEfSe; Figure 4A,B). With a default LDA cutoff of ±2.0, 19 and 15 different taxa were found in the CON group and RPF group, respectively. The bacteria biomarkers in the CON group were *norank_f_Bacteroidates_RF16_group*, *Campylobacter*, *Agathobacter*, *Peptococcus*, *norank_f_norank_o_Rhodospirllales*, *Elusimicrobium*, *Syntrophococcus*, and *Sphaerochaeta* and in the RPF group were *norank_f_Enbacterium_coprostanoligenes_group*, *norank_f_norank_o_Clostridia_ UCG-014*, *Odoribacter*, *Sanguibacteroides*, *Eubacterium_brachy_group*, *Lachnospiraceae_NK3A20_group*, and *Aeriscardovia*. 

### 3.5. Correlations between Fecal Bacteria and Short-Chain Fatty Acids

**Figure 5:**
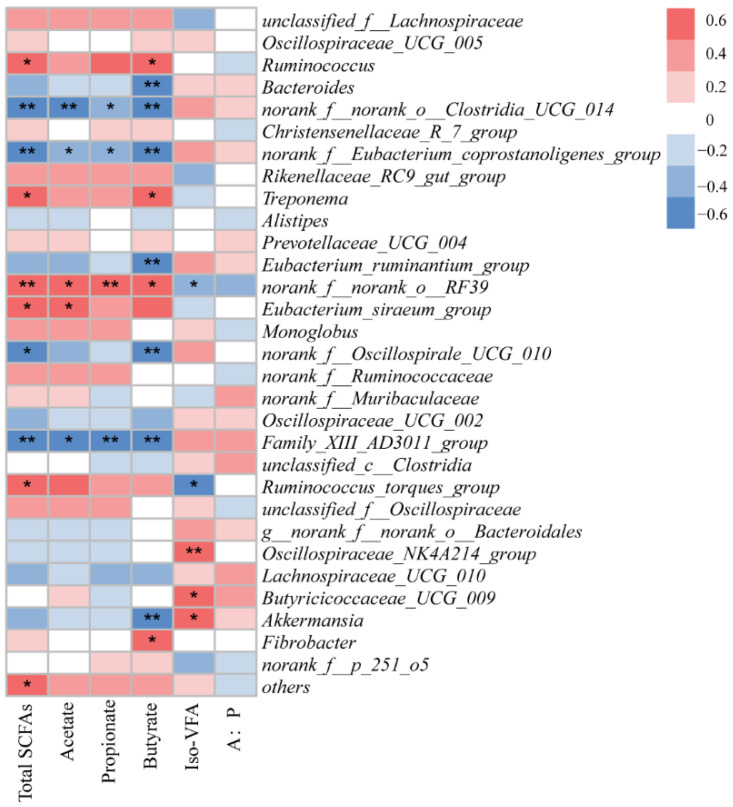
Correlation of fecal bacterial relative abundance at genus level with feces SCFA concentrations. * *p* < 0.05, and ** *p* < 0.01 according to correlation coefficient.

There were 16 positive (*p* < 0.05) and 19 negative (*p* < 0.05) correlations between the relative abundances of bacterial genera and the concentrations of SCFAs and minerals (Figure 5). *Ruminococcus* and *Treponema* were correlated positively with concentrations of fecal total SCFAs and butyrate. *Norank_f_norank_o_Clostridia_UCG_014*, *Norank_f_Enbacterium_coprostan-oligenes_group*, and *Family_XIII_AD3011_group* were negatively correlated with the concentrations of total SCFAs, acetate, propionate, and butyrate. *Oscillospiraceae_NK4A214* and *Butyricicoccaceae_UCG_009* were positively correlated with concentrations of iso-VFAs. *Bacteroides*, *Eubacterium_ruminantium_group*, and *Akkermansia* were negatively correlated with concentrations of butyrate.

## 4. Discussion

### 4.1. Effect of Dietary Rumen-Protected Fat on Growth Performance

RPFs are an important ingredient in the diets of ruminants, especially dairy cows [16]. It is well accepted that RPF benefits herd reproductive performance because they can increase energy density and reduce the risk of metabolic disorders [6,17]. Numerous studies have reported that RPF could improve the milk yield of dairy cows [6] but did not influence the ADG in Dorper sheep [10] and beef cattle [18]. However, in the present study, we found that the ADG was greater in the RPF group than in the CON group, which is in agreement with a previous study concerning finishing goats [19]. This could explain how the supplementation of RPF increased the total tract digestibility of crude protein, lipids, or crude fiber in steers [20], ewes [21], and sheep [10]. Our results showed that the final body weight was greater in the RPF group than in the CON group, which is in agreement with a previous study that reported that body weights increased in finishing beef steers [7].

### 4.2. Effects of Dietary Rumen-Protected Fat on Fecal Short-Chain Fatty Acid Concentrations

Short chain fatty acids, a type of fatty acid with fewer than six carbon atoms, are produced by gut bacteria when they ferment fiber, and they exert several effects on the host’s metabolism and immune system [22]. In the present study, we found that the concentrations of total SCFAs, acetate, propionate, and butyrate were lower in the RPF group than in the CON group, which is in agreement with a previous study on mice [23]. Acetate and butyrate are mainly generated by fiber. A previous study reported that RPF could enhance the total tract digestibility of fiber in ruminants [10]. In the present study, we found no differences in the DMI between the CON and RPF groups; that is, there was a lower fecal fiber concentration in the RPF group than in the CON group. Hence, the concentrations of acetate and butyrate were greater in the CON group than in the RPF group.

### 4.3. Microbial Community Composition in the Feces

Firmicutes and Bacteroidetes were the dominant bacterial phyla in feces, which is in agreement with previous studies on goats [24], sheep [25], dairy cows [26], and cattle [27]. Spirochaetota, including Treponema, is a phylum of double-membrane Gram-negative anaerobic bacteria regarded as a pathogenic bacterium. A previous study reported that RPF could improve starch digestibility, resulting in a lower starch content in the RPF group [19]. Furthermore, it was found that the Spirochaetota phylum and *Treponema* genus existed in starch-rich materials. Moreover, *Treponema* was associated with pectin and xylan degradation in the gastrointestinal tract [28,29]. Hence, the RAs of the Spirochaetota phylum and *Treponema* genus were lower in the RPF group than in the CON group. Furthermore, we observed that *Treponema* positively correlated with total SCFAs and butyrate. Verrucomicrobiota are widely distributed in various mammals and encode numerous carbohydrate-degrading enzymes, peptidases, and sulfatases, and their ecological functions are still poorly understood [30]. In the present study, the RA of Verrucomicrobiota was greater in the CON group than in the RPF group, and the reason behind this needs to be clarified in the future. In addition, the levels of Fibrobacterota, an important cellulose-degrading phylum, were lower in the RPF group than in the CON group, which could explain why the fecal fiber content was lower in the RPF group than in the CON group [31].

At the genus level, the most-dominant fecal bacterium was *unclassified_f__Lachnospiraceae*, followed by *Oscillospiraceae-UCG-005* and *Ruminococcus*. However, previous studies reported that the most-abundant genera in feces were bacteriodes for Hainan black goats and Saanen goats [32], Escherichia for sheep [33], and *Prevotella* for cattle [27].

This difference could be explained by the differences in diets and animal species between these studies. As for fibrolytic bacteria, such as *Ruminococcus* [34] and *Rikenellaceae_RC9_gut_group* [35], the RA of these bacteria was greater in the CON group than in the RPF group. This could be explained by the fact that the fecal fiber content was lower in the RPF group than in the CON group [30]. It was reported that *Ruminococcus* was positively correlated with cecal acetate concentrations in broilers [4]. However, we found that *Ruminococcus* strains were not correlated with acetate. This difference could be related to the types of diets or animal species. *Bacteroides* is regarded as a predictive biomarker for weight change [36]. In the present study, we found that the RA of *Bacteroides* was greater in the RPF group than in the CON group, which could be explained by the fact that a greater RA of Bacteroides indicates a greater body weight [37]. The abundance of *Norank_f_norank_o_Clostridia_UCG-014*, a beneficial bacterium, was greater in the RPF group than in the CON group, which is in agreement with a previous study on yaks [38]. In addition, we found *Norank_f_norank_o_Clostridia_UCG-014* was negatively correlated with total SCFAs, acetate, propionate, and butyrate. *Eubacterium_coprostanoligenes* could generate beneficial SCFAs and then play an anti-inflammatory role for the host. Our results showed a greater RA of *Eubacterium_coprostanoligenes* in the RPF group than in the CON group, which is in agreement with a previous study in which it was reported that a high-fat diet led to a sharp increase in the RA of *norank_f__Eubacterium_coprostanoligenes_group* [39]. Moreover, we found *norank_f__Eubacterium_coprostanoligenes _group* was negatively correlated with total SCFAs, acetate, propionate, and butyrate.

### 4.4. Correlations between Fecal Bacteria and Short-Chain Fatty Acids

*Eubacterium_siraeum_group* is a validly published species of the genus *Eubacterium*, but it may belong to a new genus based on phylogenomic analysis. A previous study reported that *Eubacterium_siraeum_group* is associated with cellulose degradation in Eospalax cansus [40]. Hence, in the present study, we found that *Eubacterium_siraeum_group* was positively correlated with fecal acetate. *Akkermansia* is often reported to facilitate butyrate, which is associated with positive health effects in the gut. Interestingly, we found that *Akkermansia* was negative correlated with butyrate. *Ruminococcus_torques_group* is a Gram-stain-positive, obligately anaerobic bacterium known to be a bile-acid-converting bacterium. Our results showed the *Ruminococcus_torques_group* was positively correlated with total SCFAs, which is in agreement with a previous study conducted using a colitis mouse model [41].

## 5. Conclusions

In the RPF group, the ADG was greater and the DMI-to-ADG ratio was lower than those in the CON group. Additionally, fecal bacteria were altered when providing supplementary RPF to the goats. The relative abundances of *Ruminococcus*, *Rikenellaceae_RC9_gut_group*, *Treponema*, *nor-ank_f__norank_o__RF39*, *Eubacterium_siraeum_group*, and *Ruminococcus_torques_group* were lower in the RPF group than in the CON group. The relative abundances of *Bacteroides*, *norank_f__norank_o__Clostridia_UCG-014*, *norank_f__Eubacterium_coprostanoligenes_group*, *Eubacterium_ruminantium_group*, *norank_f__Oscillospirale-UCG-010*, *Oscillospiraceae_UCG-002*, and *Family_XIII_AD3011_group* were greater in the RPF group than in the CON group. We conclude that the supplementation of 2.4% RPF to Leizhou goats could improve their growth performance and alter their fecal bacteria. Future studies need to investigate the nutrient digestibility and absorption effects of RPF on the rumen and hindgut.

## Figures and Tables

**Table 1:** Ingredients and chemical compositions of the diets offered to the goats.

Items	Content
Ingredients, % of DM	
Corn stalk	50.0
Corn grain (ground)	15.0
Soybean meal	13.0
Wheat bran	5.00
Barley grain	8.00
Distillers dried grains with solubles	4.15
Sodium bicarbonate	1.00
Limestone	1.20
Calcium hydrogen phosphate	0.95
Sodium chloride	0.50
Urea	0.20
Premix ^1^	1.00
Chemical composition	
DM, %	91.5
CP ^2^, %	13.5
NDF, %	41.0
ADF, %	23.5
EE, %	5.00
Calcium, %	0.76
Phosphorus, %	0.32

DM = dry matter; CP = crude protein; NDF = neutral detergent fiber; ADF = acid detergent fiber; EE = ether extract. ^1^ The premix provided the following per kg: vitamin A, 12,000 IU; vitamin D, 2000 IU; vitamin E, 30 IU; Cu, 12 mg; Fe, 64 mg; Mn, 56 mg; Zn, 60 mg; I, 1.2 mg; Se, 0.35 mg; Co, 0.4 mg. ^2^ Calculated as nitrogen × 6.25.

**Table 2:** Effect of dietary supplementation of rumen-protected fat on feed intake and growth performance of goats.

Items	CON	RPF	SEM	*p*-Values
IBW, kg	13.37	13.31	0.024	0.872
FBW, kg	16.52	16.73	0.091	0.027
DMI, g/d	906	920	20.4	0.733
ADG, g/d	75.1	87.5	3.78	0.012
DMI:ADG	12.1	10.5	0.30	0.041

CON = control group; RPF = rumen-protected fat; SEM = standard error of the means; IBW = initial body weight; FBW = final body weight; DMI = dry matter intake; ADG = average daily gain.

**Table 3:** Effect of dietary supplementation of rumen-protected fat on feces concentrations of short-chain fatty acids in goats.

Items	CON	RPF	SEM	*p*-Values
Total SCFA, Mm	14.9	13.6	0.33	<0.01
Acetate, Mm	11.3	10.5	0.29	0.025
Propionate, Mm	1.84	1.65	0.078	0.036
Butyrate, Mm	1.25	0.83	0.087	<0.01
Iso-VFA, Mm	0.57	0.65	0.044	0.117
Acetate:Propionate	6.21	6.50	0.423	0.511

CON = control group; RPF = rumen-protected fat; SCFA = short-chain fatty acids; SEM = standard error of the means.

**Table 4:** The alpha diversity in response to dietary rumen-protected fat in goat feces.

Items	CON	RPF	SEM	*p*-Values
Ace	1518	1442	40.1	0.512
Chao	1480	1414	35.2	0.226
Coverage	0.996	0.996	0.0001	0.999
Shannon	5.31	5.27	0.05	0.519
Simpson	0.013	0.015	0.001	0.792
Sobs	1360	1280	38.13	0.851

CON = control group; RPF = rumen-protected fat; SEM = standard error of the means.

**Table 5:** Effect of dietary supplementation of rumen-protected fat on fecal bacteria (at phylum level, >1.0% of total reads) in goat.

Items	CON	RPF	SEM	*p*-Values
Firmicutes	69.0	72.1	0.52	<0.001
Bacteroidota	22.9	22.6	0.26	0.610
Spirochaetota	5.80	3.32	0.411	<0.001
Verrucomicrobiota	0.48	0.65	0.033	0.003
Fibrobacterota	0.57	0.41	0.031	0.003
Others	1.34	0.90	0.074	<0.001

CON = control group; RPF = rumen-protected fat; SEM = standard error of the means.

**Table 6:** Effect of the dietary supplementation of rumen-protected fat on fecal bacteria (at phylum level, >0.5% of total reads) in goats.

Items	CON	RPF	SEM	*p*-Values
*unclassified_f__Lachnospiraceae*	12.8	12.1	0.243	0.173
*Oscillospiraceae-UCG-005*	7.84	7.55	0.228	0.561
*Ruminococcus*	7.52	6.46	0.211	<0.01
*Bacteroides*	6.12	7.27	0.240	<0.01
*norank_f__norank_o__Clostridia_UCG-014*	4.72	6.52	0.293	<0.001
*Christensenellaceae_R-7_group*	5.55	5.33	0.100	0.301
*norank_f__Eubacterium_coprostanoligenes_group*	3.78	5.93	0.338	<0.001
*Rikenellaceae_RC9_gut_group*	5.11	4.54	0.142	0.033
*Treponema*	5.77	3.30	0.393	<0.001
*Alistipes*	3.56	4.09	0.215	0.233
*Prevotellaceae_UCG-004*	2.66	2.67	0.099	0.956
*Eubacterium_ruminantium_group*	1.58	3.01	0.250	<0.001
*norank_f__norank_o__RF39*	2.83	1.72	0.201	<0.01
*Eubacterium_siraeum_group*	2.45	1.89	0.110	<0.01
*Monoglobus*	2.20	2.15	0.070	0.729
*norank_f__Oscillospirale-UCG-010*	1.74	2.57	0.155	0.001
*norank_f__Ruminococcaceae*	2.06	1.92	0.122	0.606
*norank_f__Muribaculaceae*	1.66	1.57	0.129	0.758
*Oscillospiraceae_UCG-002*	1.19	1.65	0.090	<0.01
*Family_XIII_AD3011_group*	1.01	1.68	0.126	<0.01
*unclassified_c__Clostridia*	1.18	1.10	0.084	0.648
*Ruminococcus_torques_group*	1.27	0.73	0.102	<0.01
*unclassified_f__Oscillospiraceae*	0.97	0.94	0.030	0.618
*g__norank_f__norank_o__Bacteroidales*	0.61	0.61	0.048	0.923
*Oscillospiraceae_NK4A214_group*	0.58	0.62	0.052	0.662
*Lachnospiraceae_UCG-010*	0.51	0.60	0.029	0.084
*Butyricicoccaceae_UCG-009*	0.57	0.53	0.043	0.597
*Akkermansia*	0.42	0.56	0.051	0.199
*Fibrobacter*	0.57	0.41	0.054	0.163
*norank_f__p-251-o5*	0.49	0.44	0.038	0.521
*others*	10.7	9.53	0.342	0.088

CON = control group; RPF = rumen-protected fat; SEM = standard error of the means.

## Data Availability

Data are contained within the article.
